# Analysis of vascular disruption in zebrafish embryos as an endpoint to predict developmental toxicity

**DOI:** 10.1007/s00204-023-03633-x

**Published:** 2023-12-21

**Authors:** Julia Nöth, Wibke Busch, Tamara Tal, Chih Lai, Akhil Ambekar, Tobias R. Kießling, Stefan Scholz

**Affiliations:** 1 Department of Bioanalytical Ecotoxicology, Helmholtz Centre for Environmental Research—UFZ, Permoserstraβe 15, 04318 Leipzig, Germany; 2 University of St. Thomas, St. Paul, MN USA; 3 Duke University, A.I. Health Fellow-Associate in Research, Durham, NC USA; 4 Scientific Software Solutions, 04275 Leipzig, Germany

**Keywords:** FishInspector, Cardiovascular system, Alternatives to animal testing, 3Rs, High throughput, High content

## Abstract

Inhibition of angiogenesis is an important mode of action for the teratogenic effect of chemicals and drugs. There is a gap in the availability of simple, experimental screening models for the detection of angiogenesis inhibition. The zebrafish embryo represents an alternative test system which offers the complexity of developmental differentiation of an entire organism while allowing for small-scale and high-throughput screening. Here we present a novel automated imaging-based method to detect the inhibition of angiogenesis in early life stage zebrafish. Video subtraction was used to identify the location and number of functional intersegmental vessels according to the detection of moving blood cells. By exposing embryos to multiple tyrosine kinase inhibitors including SU4312, SU5416, Sorafenib, or PTK787, we confirmed that this method can detect concentration-dependent inhibition of angiogenesis. Parallel assessment of arterial and venal aorta ruled out a potential bias by impaired heart or blood cell development. In contrast, the histone deacetylase inhibitor valproic acid did not affect ISV formation supporting the specificity of the angiogenic effects. The new test method showed higher sensitivity, i.e. lower effect concentrations, relative to a fluorescent reporter gene strain (Tg(KDR:EGFP)) exposed to the same tyrosine kinase inhibitors indicating that functional effects due to altered tubulogenesis or blood transport can be detected before structural changes of the endothelium are visible by fluorescence imaging. Comparison of exposure windows indicated higher specificity for angiogenesis when exposure started at later embryonic stages (24 h post-fertilization). One of the test compounds was showing particularly high specificity for angiogenesis effects (SU4312) and was, therefore, suggested as a model compound for the identification of molecular markers of angiogenic disruption. Our findings establish video imaging in wild-type strains as viable, non-invasive, high-throughput method for the detection of chemical-induced angiogenic disruption in zebrafish embryos.

**Supplementary Information:**

The online version contains supplementary material available at 10.1007/s00204-023-03633-x.

## Introduction

Embryonic development is a highly complex cell division and differentiation process that requires a fine-tuned interplay of morphogenic factors across diverse tissue types. Due to its crucial role to transport oxygen and essential nutrients, blood vessel formation is critical for organismal growth. Therefore, disruption of vascular development is considered to be an important mode of action in developmental toxicity, associated with prenatal loss, malformations, maternal placental complications, and undesired neurodevelopmental outcomes (D'Amato et al. 1994; Hanson and Gottesman 2005; Hoyme et al. 1990; Tideman et al. 2007).

In mammals, the vascular system is the first functional organ to develop. The development of de novo blood vessels, termed vasculogenesis, provides the main blood vessel system. Subsequently, during angiogenesis, new vessels arise by a range of processes including differentiation, migration and proliferation of endothelial cells, remodeling of the extracellular matrix, and tube formation (Isogai et al. 2001; Gore et al. 2012). Disruption of angiogenesis is defined as one of six teratogenic mechanisms associated with unwanted pharmaceutical side effects (van Gelder et al. 2010). Therefore, it is important to identify chemicals with the potential to cause disruption of blood vessels. Pharmaceuticals including thalidomide (D'Amato et al. 1994), (Therapontos et al. 2009) and environmental pollutants including haloxyfop-p-methyl, tris(1,3-dichloro-2-propyl) phosphate and rotenone (Kleinstreuer et al. 2011; McCollum et al. 2017; Zhong et al. 2019; Park et al. 2020; are known to cause vascular disruption in zebrafish. In vitro and biochemical screening approaches include assays linked to vascular disruption in early life stage zebrafish (Tal et al. 2017). A commonly used cell-based method for the investigation of vascular disruption is performed in Human Umbilical Vein Endothelial cells (HUVEC), where for example proliferation, migration and tube formation are measured. While simple to use in high throughput, this screening assay lacks the complexity of multiple, interacting cell types interacting in three dimensional space (Therapontos et al. 2009; Rezzola et al. 2014; Medina-Leyte et al. 2020). In contrast, more complex animal-based models including the chick chorioallantoic membrane, corneal neovascularization, and matrigel plug assays are widely used to identify vascular disruption yet they are costly, present ethical implications, and do not provide high-throughput screening capacity (Auerbach et al. 2003).

The zebrafish embryo provides several advantages as model system for the prediction of human developmental toxicity. It represents a complex differentiating organism, with high genetic homology (up to 70% for coding gene sequences) to humans (Howe et al. 2013), but allows for screening at a small scale similar to in vitro systems. Since pathways of relevance for developmental toxicity are conserved among vertebrates (NRC 2000), the zebrafish model can, in theory, be used to predict potential adverse effects in humans. Furthermore, genetic manipulation techniques like availability of transgenic strains, efficient gene silencing techniques, have strengthened the power of the zebrafish embryo model for the study of developmental toxicity (Nishimura et al. 2016). The formation of the zebrafish blood vessel system by vasculogenesis has been described in detail previously (Isogai et al. 2001). First blood vessels are developed within the first day of development, followed by formation of intersegmental blood vessels (ISVs) via sprouting angiogenesis within 36 hpf (hours post fertilization) (Isogai et al. 2001). After 48 hpf, blood circulates through a series of aortic arteries and flows into an anterior and posterior circulatory loop. The ISVs in the trunk and tail of the fish are fully formed and functional after 72 hpf. Given their anatomical simplicity as well as the regular ordered formation of ISV (Blum et al. 2008), we propose that they can be used as an endpoint to predict chemical-induced developmental toxicity.

Various techniques have been established to visualize and investigate the cardiovascular system in zebrafish (Rosa et al. 2022) including endothelial specific reporter lines (e.g., Tg(fli1a:EGFP)^y1^, Tg(kdrl:EGFP)^s843^, Tg(fli1a:EGFP; kdrl:ras-cherry)), staining methods, and dye injection (Jung et al. 2016). The use of staining methods involves multiple working steps and can limit high-content assessments. While the use of fluorescently labeled transgenic lines has been employed (McCollum et al. 2017; Tal et al. 2014), homozygous line maintenance can be a burden on smaller fish facilities. Furthermore, requirement of transgenic lines may result in the need to assess many transgenic lines in screening approaches targeting multiple endpoints. There is a need for microscopy-based imaging techniques that do not require specific labels and can quantify vascular disruption in wildtype larvae, using basic bright filed imaging approaches. Therefore, this study aimed at developing a video-subtraction-based screening approach to identify interaction with angiogenesis in zebrafish embryos. The approach expands a previous attempt (Puybareau et al. 2015) for visualization and quantification of morphological effects using an automated, deep learning-assisted annotation software (FishInspector 1.70), in combination with KNIME workflows to derive effect concentrations for specific morphological changes. We selected four known tyrosine kinase (TK) inhibitors targeting the kinase domain of the vascular endothelial growth factor receptor 2 (VEGFR2). The TK inhibitors served as model compounds to establish the video subtraction method and to identify a model compound for subsequent molecular studies. Valproic acid (VPA) was used as a negative control to assess the specificity of anti-angiogenic effects.

## Materials and methods

### Chemicals and standard solutions

SU4312, SU5416, Sorafenib, PTK787 and valproic acid (VPA) were purchased from Sigma-Aldrich with a purity range of > 98% (Table 1).

**Table 1:** Chemical properties of tyrosine kinase inhibitors

Inhibitor name	Targets	CAS RN	Purity (%)	Log *K*_ow_	Log *D*_lipw_ at pH 7.4	Molecular weight [Da]
SU4312	VEGFR2, PDGFR, EGFR, HER-2, IGF (Sun et al. 1998)	5812-07-7	98	3.26	3.73	264.32
SU5416	VEGFR2, PDGFR, FIt-1, FIt-4, c-kit (Haddad 2012)	204005-46-9	98	3.06	2.09	238.28
PTK787	VEGFR2, VEGFR-1, PDGF, FIt-4, and c-Kit (Gotink and Verheul 2010)	212141-51-0	98	4.98	0.78	419.73
Sorafenib	VEGFR, PDGFR, FGFR1, KIT, RAF (Gotink and Verheul 2010)	284461-73-0	98	5.30	5.53	464.82
Valproic acid	HDAC1 (Phiel et al. 2001)	99-66-1	98	2.75	1.9	144.21

References for the observation of inhibition and mechanism of action are provided within the “Target” column. *D*_lipw_ values were obtained as described in Klüver et al. (2019)

Stock solutions were prepared in 100% dimethyl-sulfoxide (DMSO) and diluted in exposure medium as specified in ISO 7346-3 (1996) [80 mM CaCl_2_·2H_2_O, 20 mM MgSO_4_·7H_2_O, 31 mM NaHCO_3_, 3.1 mM KCl]. The final concentration of DMSO was 0.01% (v/v). The pH of the highest test concentration was adjusted to pH 7.4 and all other test concentrations were established by dilution of the highest concentration.

### Zebrafish culture and fish embryo production

A wild-type adult zebrafish strain (*D. rerio*, strain OBI/WIK, generation F3), originating from an in-house cross of zebrafish strains UFZ-OBI and WIK, was used for all experiments. The Tg(kdr:EGFP) strain was kindly provided by the University of Jena. Fish were fed twice daily with dry food (SDS-400, Special Diets Services, Essex England) and once daily with *Artemia sp*. Fish were cultured under 14h light/10h dark photoperiod in 20 L aquaria with 1–2 fish per liter density (26.5 ± 1 °C). Water quality parameters were pH 7–8; water hardness 2–3 mmol/L, conductivity 540–560 S/cm, nitrate < 2.5 mg/L, nitrite < 0.025 mg/L, ammonia < 0.6 mg/L, oxygen saturation 87–91%. Spawning trays were inserted 4–6 h before the end of the light cycle. The following day, spawning was initiated by the onset of light and eggs were collected within 1 h. Exposure in all experiment was ceased at 96 hpf. Facilities for breeding and the production of embryos were licensed by Landesdirektion Leipzig (Aktenzeichen 75-9185.64).

### Chemical exposure

Fertilized and normally developed embryos were selected according to Kimmel et al. (1995) with a dissection microscope (Olympus SZx7-ILLT) at 1 hpf. Depending on experiment, exposure was started within 2–3 h post-fertilization (16–128 cell stages) or at 24 hpf. Exposure was conducted in rectangular 96-well microplates (Clear Polystyrene, flat bottom, Uniplate, Whatman, GE Healthcare) covered by a lid with a test volume of 400 µL of exposure media. For each replicate one embryo per well and 16 wells per concentration were assessed. Experiments were performed with at least two replicates on independent days and breeding.

### Assessment of sensitivity ratio

Baseline toxicity was calculated using the *D*_lipw_ and a zebrafish embryo-specific QSAR (Klüver et al. 2019). Baseline toxicity and observed mortality were used to calculate two sensitivity ratios (SRs), SR_baseline_ (= predicted baseline toxicity LC_50_/IC_50_) and the SR_mortality_ (= observed LC_50_/observed IC_50_). In case that no mortality was observed, the maximum test concentration was used to obtain the minimal SR. Similar to previous work (Lee et al. (2022)) we considered compounds and exposure conditions with an SR_baseline_ > 10 and SR_mortality_ > 4 as indicators of specific effects.

### Model training for high-throughput imaging of zebrafish embryos

Automated imaging of zebrafish developmental phenotypes was conducted with the VAST BioImager (Union Biometrica, Gees, Belgium (Pardo-Martin et al. 2010; Pulak 2016)) equipped with a large particle sampler (LP sampler, Union Biometrica). Lateral and dorsoventral images were obtained at 10 µm resolution at 96 hpf. Prior to imaging, all embryos were anesthetized by adding 20 µl tricaine solution (6 mg/l tricaine, TRIS 26 mM, pH 7.5 ± 0.1) to each well of the microplate. Images were processed with an amended version (1.70, available via https://codebase.helmholtz.cloud/ufz/tb3-cite/biotox/FishInspector) of the FishInspector imaging software (Teixidó et al. 2019). The FishInspector software allows to automatically annotate morphological features including body contour, eye, lower mouth tip, pericard, otoliths, notochord, swimbladder, and yolk and to calculate diverse metrics such as feature length, size, curvature indices, or angle between feature-connecting lines. In contrast to the previously published version, FishInspector 1.70 detects morphological features based on a deep learning approach using the Matlab Neural Network Toolbox and Matlab Image Processing Toolbox (Mathworks, Natick, MS). In addition, metric calculation was included into the software, and meta information such as test compound, exposure window or test concentration was associated to each image using a microplate layout file. To facilitate training of the models, two additional programs were generated in Matlab including FishTrainer 0.9 (for training of semantic segmentation models of structural features) and FishClassificator 0.9 (for training of position classes to be used in automated flipping of images) available via same repository as FishInspector software. For image training, image size was adjusted to 450 × 300 pixel to reduce computing time. This resolution was sufficient to detect the selected features (data not shown). For training of the deep learning models, approximately 3000 previously annotated images (Teixidó et al. 2022) were used. The automated annotations were corrected by the user in the event that they were not adequately detecting visible structures. User-bias was avoided by a blinded assessment of images. Following model training, experimental images used for annotation had a resolution of 1024 × 190 pixels.

### Automated high-throughput image analysis of zebrafish embryos

FishInspector provided a JSON file corresponding to each image file containing the coordinates of all features and the applied smoothing factor. All images and videos used for this publication can be found at the BioImage Archive S-BIAD954 (https://www.ebi.ac.uk/biostudies/bioimages/studies/S-BIAD954). For each image, a csv file was generated containing calculated metrics. The software automatically provides a summary table containing metrics r of individual embryos. All experimental images and corresponding csv files can be found in Supplementary Material. All subsequent analyses were conducted using the summary excel file and a KNIME workflows with embedded R-scripts, as previously described (Teixidó et al. 2022). The KNIME workflow (all workflows are available via https://git.ufz.de/automated-fish-embryotest/zebrafish-embryo-crc/zebrafish-isv) converted pixel metrics into µm or µm^2^, combined results from different replicates, and conducted concentration response analysis to calculate effect concentrations (EC_10_ and EC_50_). Concentration–response modeling was performed using the drc R package (Ritz et al. 2015). The analyzed file includes endpoint names based on the FishInspector software. Metrics were translated to ontologies available in Ontobee (https://ontobee.org/) (Supplement Table 1). When an appropriate term was missing in Ontobee, it was generated using existing terms for object class, property and qualifier. If two FishInspector endpoints shared the same ontology, the most sensitive was selected based on corresponding EC_50_ values.

### Determination of heart rate

During the VAST imaging process, a video of 15 s at 30 frames per second was recorded for each embryo in lateral position for subsequent video-based determination of the heart frequency. Quantification of the heart rate was performed as previously described (Teixidó et al. 2022). Only embryos with a detected heartbeat were evaluated. A Windows standalone GUI based on a Matlab script was used to extract heart rates and conduct concentration–response modeling.

### Quantification of angiogenesis in zebrafish embryo

To determine the number of ISVs in the trunk region, high-resolution images of 96 hpf-old zebrafish embryos were recorded using a Leica DM6B-Z microscope (Leica, Wetzlar, Germany) attached to the VAST imaging system, and a camera (Leica DFC9000 sCMOS, objective: HC APO L U-V-I 20x/0.50 WATER UV). A series of 20 images with a frame rate of 20 images per second and a 2 × 2 pixel binning was recorded in autofocus mode for each embryo. For the Tg(kdr:EGFP) strain, one additional fluorescence image was obtained using a fluorescent filter set (emission wavelength 540 nm, excitation filter BP 450–490) and a camera exposure time of 129 ms and a 2. × 2 binning in autofocus mode.

A series of MATLAB scripts (Supplement File ‘Workflow 3.m’ and ‘Workflow 4.m’) was used to convert the image series into a video and to visualize the blood vessel by applying a video subtraction approach (Figure SI1). Briefly, each video frame was subtracted from the average image of the whole image sequence and converted to a binary image by applying a Gaussian blur (*σ* = 5) and a threshold of 0.025 to obtain optimal contrast in binary images. Subsequently, the sum of all binary images was calculated. Due to movement of the blood cells the subtraction approach removed all parts from the image which were not moving resulting in an image of the blood vessels. This binary image of the blood vessel network in the zebrafish embryo tail was then processed with the FishInspector annotation software (version 1.70). Due to the lack of previously annotated images, no training models were available, and hence, labelling was conducted manually, by positioning a 113pixel diameter circle. The number of ISVs was obtained by application of a KNIME workflow to FishInspector annotation files. Subsequently concentration–response analysis was conducted using a KNIME workflow. The coefficient of variation (CV) of controls was calculated for both the subtraction and the fluorescence-based method by dividing the standard deviation of individual embryos with the control mean:

$${\mathrm{CV}}_{\mathrm{Control}}= \frac{\mathrm{SD}}{\mathrm{Mean}}.$$

The analysis was performed by combining the results of at least two independent replicates.

### Concentration–response modeling

Concentration–response curves were fitted for lethality, heart rate, morphological features as previously described (Teixidó et al. 2019, 2022). The assessment of lethality and morphological features was based on the percentage of embryos affected. Therefore, morphological endpoints were dichotomized, i.e. feature metrics were converted to the percentage of embryos deviating from controls. An embryo was considered to deviate when it was outside 1.5-fold of the standard deviation range of the mean of the control group. The control mean was determined by pooling control embryos across all experiments (*n* = 160) (available at git.ufz.de). For heart rate and angiogenesis (number of ISVs) the analysis was based on normalized values, i.e. for each experiment the values of the treatment were divided by the control value. The experiments were designed to calculate effect concentrations based on modelled curve fits. Different concentrations were used in at least two independent replicates, with means of each replicate based on the exposure of 16 different embryos per concentrations. Effect concentration were only modeled when three conditions were met. (1) The Tukey trend test based on the R package "tukeytrend" (Schaarschmidt et al. 2022) was deriving a maximum p-value of 0.05. (2) The Akaike information criterion (AIC) for the log-logistic model was lower than the AIC for a linear model with slope 0 (lower *p* values for the letter would indicate that there is no trend in the data). (3) We calculated effect concentrations only if at least 30% of embryos for that endpoint were affected in at least one exposure concentration of (for normalized data) a reduction or increase by 30% was observed. This criterion was used to account for variable features (with 10–20% random effects in controls), and to avoid artefacts by extrapolation to EC_50_ concentrations when the maximum effect was below 50%. Visual inspection of concentration–response curves indicated that the combination of these criteria led to the automated modeling only for data sets with a clear concentration–response relationship. For all endpoints that did not meet the criteria, it was considered that there is no effect by the exposure.

## Results

### Visualization of the vascular network by subtraction of video frames

**Fig. 1:**
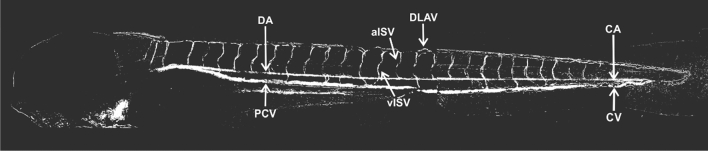
Angiogram obtained by video frame subtraction of a zebrafish embryo at 96 hpf, compiled from nine separate and overlapping images obtained with a 20 × objective. *DA*—dorsal aorta, *PCV*—posterior cardinal vein, *aISV*—arterial intersegmental vessel, *vISV*—venous intersegmental blood vessel, *DLAV*—dorsal longitudinal anastomotic vessel, *CA*—caudal artery, *CV*—caudal vein

Due to the movement of blood cells, the vascular anatomy of the developing zebrafish embryo can be visualized by subtraction of video frames. Structures in the trunk region can be easily assessed as the region is mostly transparent at 96 hpf (Fig. 1). For the assessment of effects on angiogenesis, we selected 96 hpf given that the development of the cardiovascular system is quite advanced at this stage and various types of structures are visible by video frame subtraction including the caudal aorta (CA), caudal vein (CV), posterior caudal vein (PCV), venous and arterial intersegmental vessels (vISV and aISV), and to a limited extent, dorsal longitudinal anastomotic vessels (DLAVs). Vascular structures located in the trunk at this stage are difficult to analyze by the image subtraction approach due to shading by pigmentation. Qualitative inspection of all subtraction images indicated that vascular structures (DA, CA, PCV and CV) were not affected in any of the treatments. Therefore, and given that the focus of the work was on angiogenesis, we investigated ISVs in all subsequent analyses.

### Assessment of angiogenesis inhibition by the subtraction images is more sensitive compared to fluorescence-based imaging technique

**Fig. 2:**
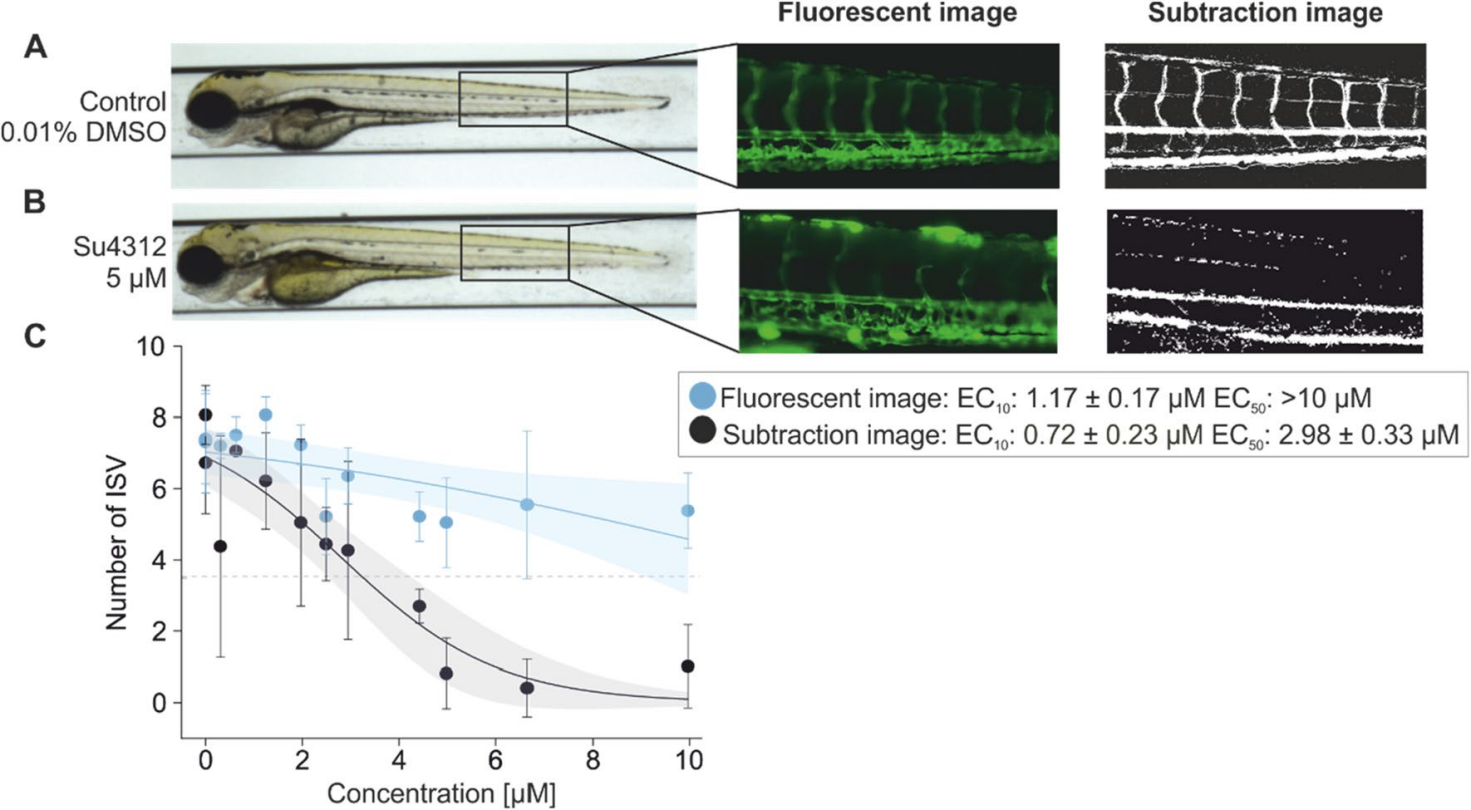
Subtraction images represent the function of ISV and therefore are a more sensitive endpoint. Embryos of the transgenic strain Tg(KDR:EGFP) were exposed to (**A**) vehicle (0.01% DMSO) or (**B**) 0.31–10 µM SU4312 at 24 hpf. At 96 hpf, larvae were visualized. The tail region of embryos was imaged using both fluorescence microscopy and the video subtraction method to compare the detection of ISVs between the two methods. **C** Concentration–response curves for the number of ISVs represented for both visualization techniques indicate more sensitive effect concentration values using the subtraction method (EC_10_ Fluorescent technique: 1.17 µM, EC_50_ Subtraction technique: 2.98 µM) (*N* = 16 each concentration, 2 replicates)

To establish the subtraction-based method and to compare it with frequently used approach to visualize the cardiovascular system in zebrafish, the transgenic line Tg(kdr:EGFP) with GFP-labeled endothelial cells was exposed from 24 to 96 hpf to a series of concentrations of 0.31–10 µM SU4312. ISVs were analyzed using the fluorescent images as well as the video subtraction approach. Comparing the CV of controls for the number of ISV between both methods indicated a slightly higher variability of the subtraction approach (coefficient of variations of 0.20 versus 0.19). This was mainly due to some control samples for which fewer ISV were detected. (SI 2). For both approaches, analysis of ISV number after SU4321 treatment in transgenic embryos showed a concentration-dependent reduction of ISVs. However, due to weaker effects, no IC_50_ value could be obtained for fluorescence-based analysis, while the calculated IC_50_ of the subtraction-based analysis was 2.98 µM (Fig. 2C). The IC_10_ values for ISV reductions differed by a factor of 1.6 (1.17 µM for fluorescent image technique and 0.72 µM for subtraction image technique, respectively) (SI 3). Albeit the confidence intervals of the subtraction method were slightly higher, due to the higher sensitivity with regard to the modelled effect concentration, the video subtraction approach using wildtype strains was used for all subsequent experiments. We also compared whether differentiation between vISV and aISV would reveal differences in sensitivity and variability (SI 4). A combined assessment of the ISV showed a lower variability and revealed lower effect concentrations (all ISVs EC_50_ 2.98 ± 2.98 µM, vISVs EC_50_ 0.75 ± 0.17 µM and aISVs EC_50_ 2.58 ± 3.12 µM).

### Exposure to tyrosine kinase inhibitors specifically reduces ISV number relative to lethality

**Fig. 3:**
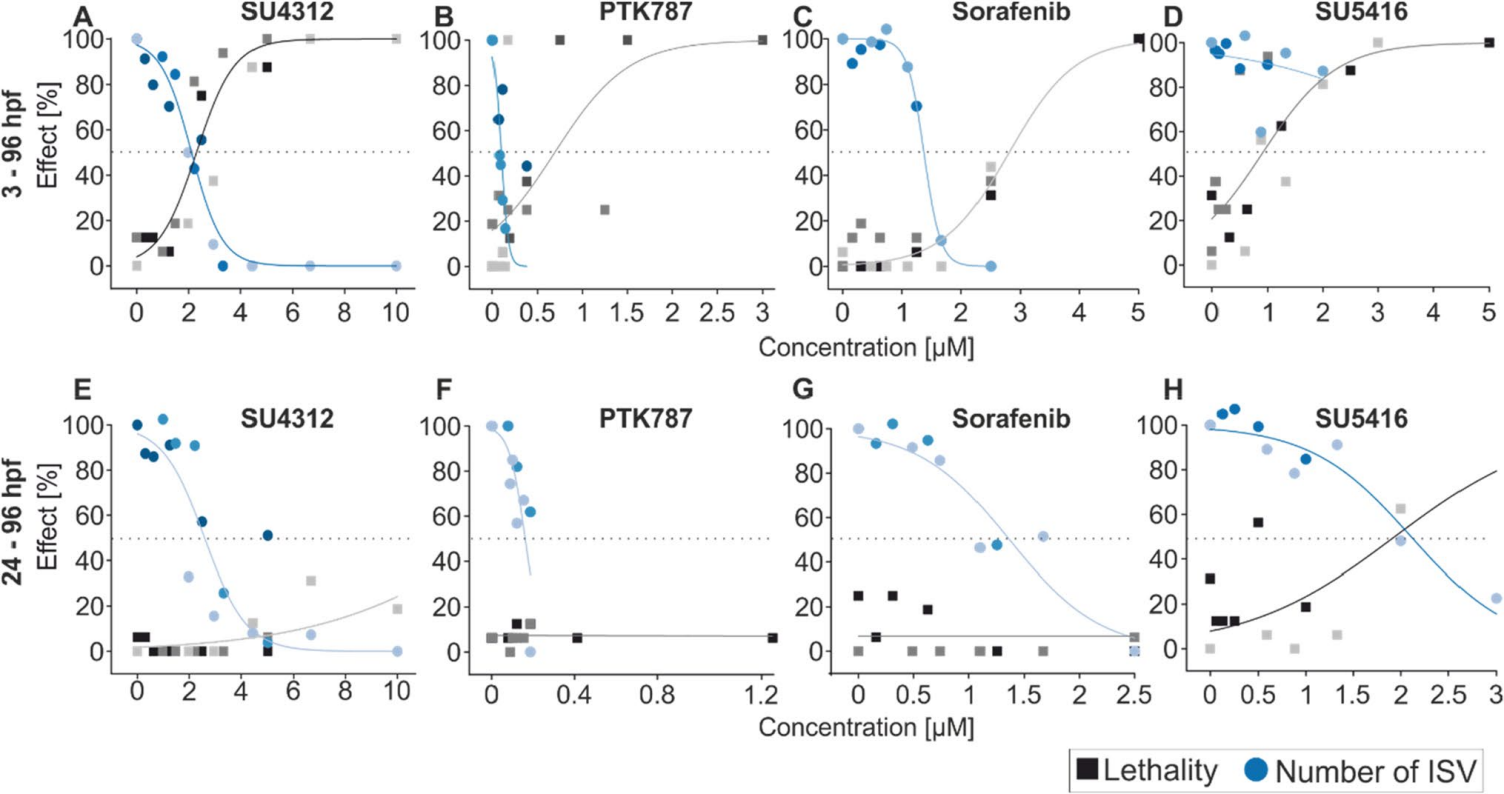
Concentration–response curves of tyrosine kinase inhibitors for mortality and ISV (intersegmental vessel) reduction following 3–96 hpf (**A**–**D**) or 24–96 hpf (**E**–**H**) exposure windows. Wildtype embryos were exposed to 0.31–10 µM SU4312 (**A**, **E**), 0.08–3 µM PTK787 (**B**, **F**), 0.16–5 µM Sorafenib (**C**–**G**), or 0.06–5 µM SU5416 (**D**–**H**). At 96 hpf, percent effect for mortality (grey) and decreasing number of ISVs (blue) was calculated. *N* = 16 and changes in color represent replicate experiments. Dotted line indicates 50% effect (colour figure online)

To identify experimental conditions with high specificity for angiogenesis effects, concentration–response relationships were generated for four known anti-angiogenic tyrosine kinase inhibitors including SU4312 (0.31–10 µM), SU5416 (0.06–5 µM), Sorafenib (0.16–5 µM) and PTK787 (0.08–3 µM), following exposure from 3–96 or 24–96 hpf. Effect concentrations were compared to those that provoked lethality or malformations in 96 hpf zebrafish embryos (Fig. 3).

Embryos exposed from 3 hpf revealed a concentration-dependent mortality with a steep increase (Fig. 3A–D, grey line). In contrast, embryos exposed from 24 hpf (Fig. 3E–G, grey line) exhibited no (SU4312, PTK787 and Sorafenib) or reduced (SU5416) mortality (Table 2). For all chemicals except SU5416, IC_50_ values for decreasing ISV number were not affected by exposure window, indicating a specific effect on ISV sprouting angiogenesis. In the case of SU5416, an ISV IC_50_ could not be obtained following chemical exposure from 3 to 96 hpf exposure due to increased mortality (Fig. 3D). Furthermore, only exposure to SU5416 induced lethality in the later exposure scenario (Fig. 3H, Table 2) and the confidence intervals of LC_50_ and IC_50_ do overlap indicating no statistically significant difference between effect concentration for mortality and ISV inhibition.

**Table 2:** IC_50_ values of ISV inhibition for early and late exposure window

TKI compound	3–96 hpf exposure		24–96 hpf exposure		Predicted baseline
	LC_50_ (µM)	IC_50_ (µM)	LC_50_ (µM)	IC_50_ (µM)	LC_50_ (µM)
SU4312	2.11 ± 0.22	2.30 ± 0.42	NA	2.59 ± 0.60	33.5
PTK787	0.68 ± 0.51	0.09 ± 0.04	NA	0.14 ± 0.03	29,171
Sorafenib	2.80 ± 0.31	1.37 ± 0.1	NA	1.36 ± 0.24	0.55
SU5416	0.89 ± 0.42	NA	1.93 ± 0.83 µM	2.10 ± 0.35	1395

The predicted baseline toxicity refers to the LC_50_ of fish embryos. Numbers indicate the modelled effect concentration ± 95% confidence intervals

**Fig. 4:**
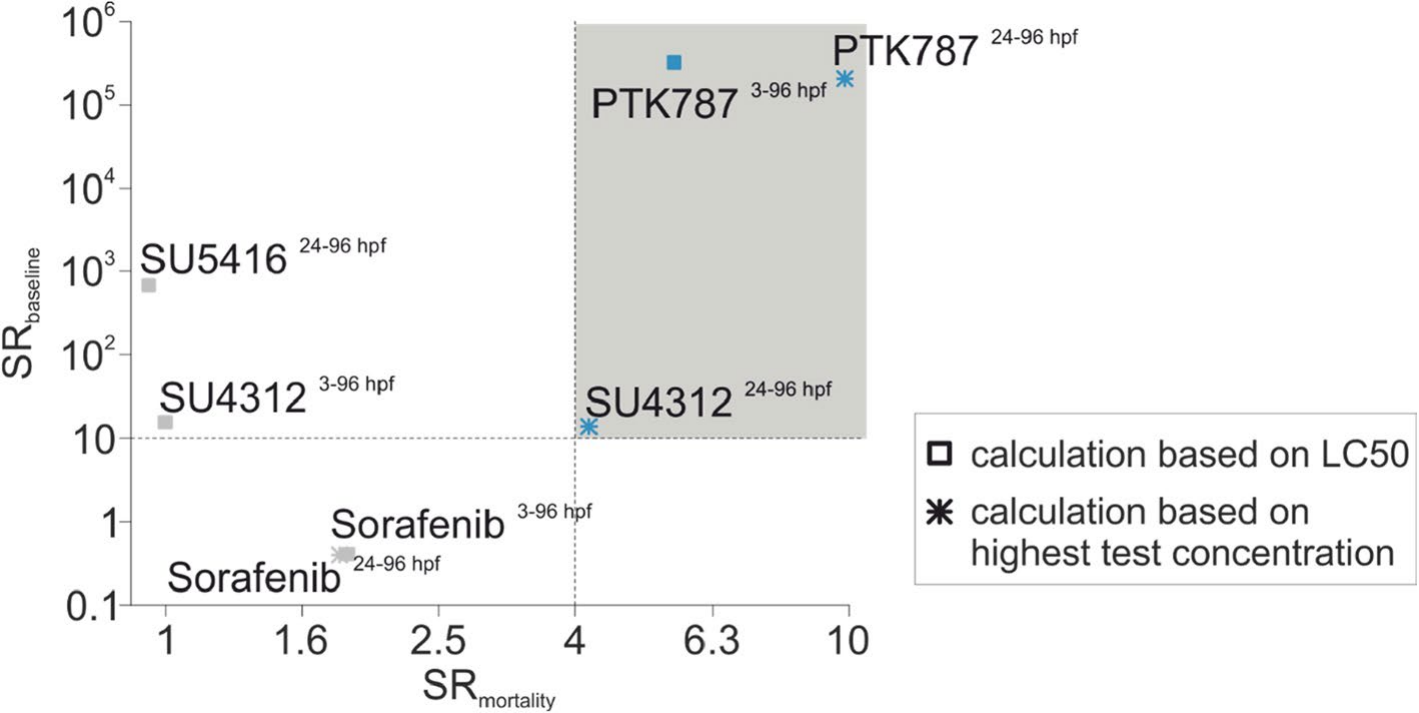
Exposure to PTK787 or SU4312 specifically decreased ISV number. Sensitivity ratios (SRs) of anti-angiogenic responses indicated by comparison of SR_baseline_ (baseline toxicity/IC_50_) and SR_mortality_ (LC_50_/IC_50_). If no mortality was observed, the maximal test concentration was used to calculate the ratio, representing the minimal ratio (indicated by an asterisk). Thresholds of 10 (SR_baseline_) and 4 (SR_mortality_) were used to indicate specificity. Compounds and exposure scenarios where both thresholds were exceeded are indicated by the grey shaded area (colour figure online)

To assess compound specificity for reduction of ISVs, effect concentrations for anti-angiogenesis were also compared to the observed LC_50_ and the predicted baseline toxicity LC_50_ which represents the predicted unspecific lethal effect concentration based on the hydrophobicity of a compound and interactions with cellular membranes (Lee et al. 2022). Values above a threshold of 4 (SR_mortality_) and 10 (SR_baseline_) were used to indicated specificity of the observed effects on angiogenesis (thresholds are indicated by dotted lines in Fig. 4). The highest specificity was observed for concentrations of early PTK787 and late exposure of PTK787 and SU4312 (Table 2).

### Angiogenesis inhibition is observed below concentrations causing other developmental disorders

**Fig. 5:**
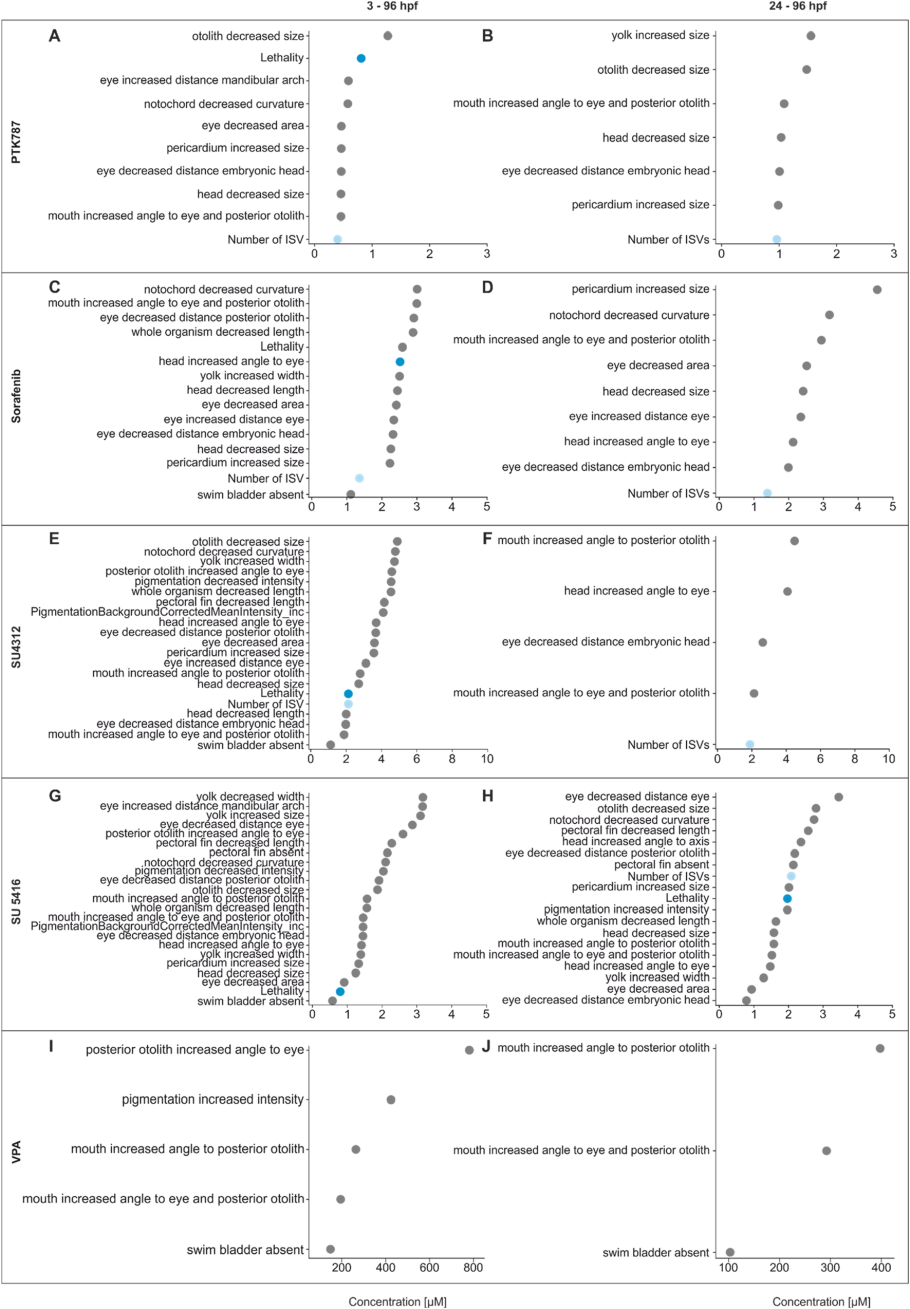
Developmental toxicity profile of tyrosine kinase inhibitors at 96 hpf based on automated image analysis and translated to ontology terms. Dot plots represent EC_50_ and LC_50_ values for embryos exposed to SU4312, SU5416, Sorafenib, or PTK787 from 3 to 96 hpf or24-96 hpf. VPA is included as a negative control. Data points corresponding to reduction of ISV are highlighted in bright blue and the LC_50_ in dark blue. For a comparison of the similarity and overlap of the morphological effects, see supplemental Fig. SI 7 (colour figure online)

Similar to lethal effects (Fig. 3), morphological effects were more severe in embryos exposed from 3 to 96 hpf (Fig. 5A, C, E and G) relative to 24 to 96 hpf (Fig. 5B, D, F and H). For all compounds except SU5416, a reduction in ISV number was the most sensitive endpoint detected in embryos exposed from 24 to 96 hpf. The highest number of effected morphological endpoints was observed in SU5416 exposed embryos (Fig. 5G-H, SI 1). Exposure to SU4312 induced a high number of malformations in early-, relative to late-exposed embryos (Fig. 5E, F). Overall, exposure to the four tyrosine kinase inhibitors did not cause a specific pattern of malformations although exposure to all inhibitors caused increased pericard size (Fig. 5). Heartrate was evaluated as an additional indicator of potential cardiotoxicity (SI 5). The mean heartrate of controls (207 bpm) were in line with previously described heart rates of 96 hpf zebrafish embryos raised at 28 °C (Luca et al. 2014). No concentration-dependent trend in the change of heart rate was observed. For comparison and assessment of specificity, VPA was analysed as a negative control compound. No effect on ISV formation was observed within the tested concentration range (up to 800 µM) (SI 6). However, craniofacial malformations were observed, indicating teratogenicity (Fig. 5I–J).

## Discussion

While various in vitro tests have been developed to investigate anti-angiogenic processes, a more comprehensive assessment can be obtained using the zebrafish embryo (Auerbach et al. 2003). Therefore, transgenic lines with an endothelial marker have historically be used to visualize the cardiovascular system in developing zebrafish (Jung et al. 2016). This strategy is based on fluorescent proteins or their corresponding genes, respectively, expressed under the promotor of endothelial genes and the assessment of vessel formation based on fluorescence labeling of endothelial cells. Using subtraction images, however, the blood vessel formation can be demonstrated as well including also functional aspects and avoiding the need for transgenic strains. Previously published video-subtraction based approaches tried to target this issue. For instance, Puybareau et al. (2015) established an image-based method using blood cells in aorta and heart in medaka embryos to assess heart function parameters. This approach was also able to visualize the blood vessel network. Others visualized the vascular system by advanced technologies such as coupled optical coherence tomography and photoacoustic (OCT-PAM) microscopy. Due to a doublemutant zebrafish strain with reduced melanophores and iridophores, they were also able to visualize the blood flow in pigmented tissues such as the eye retina (Haindl et al. 2020; Deng et al. 2021). Another approach used phase variance optical coherence tomography (pvOCT) to generate 3D images of the vasculature (Chen et al. 2016). The limitation of this approach was the dependency on the detection of slow-moving blood cells. Notably, most of the aforementioned imaging techniques required very special equipment not available as ready-to-use commercial systems and lacked automated assessment for screening purposes. Furthermore, none of the visualization approaches have been studied in the context of screening and detection of chemical effects. Here, we applied automated imaging paired with quantitative assessment of chemically induced inhibition of blood vessel development. This work shows that the subtraction-based video assessment can be implemented in an automated imaging workflow and is, therefore, suitable for screening chemicals for anti-angiogenic effects. Given that the subtraction method is based on the assessment of blood flow, the higher sensitivity may already indicate functional effects (e.g. incomplete tabulation or too narrow diameter). It can be excluded that this higher sensitivity is reflecting a parallel effect on blood cell development, as the aorta and vein are shown in subtraction images, indicating the formation and flow of blood cells. Even more to highlight is that the subtraction method may capture functional impairment of angiogenesis before it is morphologically visible. It must be noted, however, that this method is only applicable when flowing blood cells are present. A lack of heartbeat or blood cell development would interfere with the assessment of angiogenesis and may overestimate antiangiogenic effects. However, this would be indicated by the reduced or no heart rate and the lack of arterial and venal vessels in the subtraction image. The subtraction method also allows to differentiate vISVs and aISVs and quantify chemical-induced effect on the formation of either ISV types (SI 4). The separate assessment of vISV and aISV for SU4312 did not reveal a different sensitivity between the two endpoints. Furthermore, a combined assessment showed a lower variability and higher sensitivity (SI 4). ISVs in the trunk region could not be assessed since pigmentation in this region compromises the visibility of blood flow and the subtraction analysis. Mutants with reduced pigmentation or advance imaging approaches with confocal optics could be used in future work to consider angiogenesis effects in the trunk region.

The selected test compounds were known antagonists of VEGFR2 (Gotink and Verheul 2010; Haddad 2012; Sun et al. 1998). However, morphological effects and the ability of the compounds to specifically trigger angiogenic defects under different exposure conditions had not been previously studied. We demonstrated that the video subtraction method was able to identify exposure conditions with high specificity for angiogenesis inhibition. This was achieved by combining the assessment of blood vessel formation with evaluation of heart rate and morphological changes. For the model compound SU4312, the video subtraction-based approaches showed a slightly higher variability when compared to an assessment based on fluorescent strain 0.20 versus 0.19, respectively), higher sensitivities (i.e., EC10 and EC50 values) were obtained by the subtraction approach (EC_50_ 2.98 µM for the video vs EC_50_ > 10 µM for the fluorescent method). Based on the increased sensitivity for the detection of angiogenesis inhibition, the subtraction method was, therefore, used for all subsequent assessments. We also included VPA as a negative control substance to verify that reduction of ISV is not an unspecific toxicity effect caused by diverse mechanisms of action. VPA is widely used to treat epilepsy and bipolar disorders and inhibitions of the histone-deacetylase is considered to be the main mechanism of action (Phiel et al. 2001). Furthermore, VPA is classified as a teratogenic substance causing cleft palate (Phiel et al. 2001). The quantitative assessment of morphological malformations indicated changes in head and jaw structures. At the same concentration range, no inhibition of ISVs formation was observed.

Exposure window is an important variable influencing the effect of chemicals on early development in zebrafish embryos (Jakobs et al. 2020; Kühnert et al. 2017). Therefore, two exposure windows were used to compare the effect of VEGR inhibition on developmental toxicity and angiogenesis. It was hypothesized that initiating the exposure shortly after fertilization may result in numerous developmental phenotypes that may hamper the detection of specific effects on blood vessel formation. Initiating the exposure at later stages may consequently reduce other developmental effects and increase the specificity of the response. Given that heart beat and blood circulation can be first observed around 24–28 hpf (Isogai et al. 2001), exposure start times of 2 and 24 hpf were selected to determine whether increased selectivity for ISV effects could be observed with a later exposure start time. As predicted, early exposure to VEGF receptor inhibitors triggered lethality and increased malformations, likely because many elements of the VEGF pathway are also involved in developmental patterning (Vieira et al. 2010) and that test compounds can bind to other kinases including PDGFR, EGFR (Gotink and Verheul 2010), all of which are involved in vertebrate development. After 24 hpf, the basic zebrafish body plan is established and this likely explains why late exposure yielded less severe phenotypic effects and a higher specificity for impaired angiogenesis.

To assess the chemical specificity for ISV impairment, the effect concentration was compared to predicted baseline mortality, observed mortality and other morphological alterations. Baseline toxicity represents the unspecific intrinsic minimal toxicity of a compound based on cellular membrane interaction and can be predicted by the hydrophobicity of a compound (e.g. as described by the liposome water partition coefficient) (Escher and Hermens 2002). Effects at or near the predicted baseline toxicity indicate that the observed effect may represented unspecific secondary responses triggered by hydrophobicity-related cytotoxicity. Using the comparison to baseline toxicity and observed mortality, chemical specificity was ranked as PTK787 > SU4312 > Sorafenib > SU5416.

A variety of malformations were observed for all test compounds, but only 2–5 types of malformations were shared between exposure of test compounds or exposure windows, respectively increase in pericard size, angle of mouth to eye and posterior otolith, and decreases in head size, eye area, eye distance to embryonic head (SI: 7). This overlap can potentially be explained due to a lack of selectivity for VEGFR2 and different degrees of binding to other kinases. Tyrosine kinases exhibit highly structural similarities especially in the ATP-binding pocket, and are involved in various developmental processes including cell proliferation, differentiation, migration, or metabolic changes (Miyazawa 2011; Hubbard and Till 2000). One possibility explaining discordant malformations across test compounds with the same mode of action could be chemical specific off-target effects. In embryos exposed beginning at 3 hpf, one of the most sensitive malformations was a lack of swim bladder inflation. This occurred following exposure to all test compounds except PTK787. Development of a functional swim bladder requires blood circulation (Yue et al. 2015). Therefore, impaired swim bladder inflation observed in embryos exposed to SU4312, SU5416 and Sorafenib may represent a secondary effect caused by inhibition of angiogenesis. However, it is also the most sensitive endpoint of VPA treatment and many other chemicals (Teixidó et al. 2022), which does not supporting a direct link of swimbladder inflation to angiogenesis. Interestingly, the effect on swim bladder inflation was not observed when exposure was started at 24 hpf. Therefore, the lack of a swimbladder could also represent other interactions with early development and differentiation processes.

Our data confirmed previously reported malformations and ISV inhibition in early life stage zebrafish (Tal et al. 2014). For PTK787, an EC_50_ for decreased ISV length was observed at 0.17 µM in previous studies (Tal et al. 2014), while in the current study a loss of ISV number at 0.14 µM was observed. In the case of SU4312, comparable IC_50_ values were reported previously (1.8 µM; Tal et al. 2014) and here (0.09 µM). For Sorafenib a scoring system of different ISV length was used to calculate an IC_50_ of 0.53 µM (Lin et al. 2019) which was comparable to the IC_50_ value reported in the current study (1.36 µM). For embryos exposed to SU5416, a previous study described an inhibition of ISV formation at 1 µM (Serbedzija et al. 1999) similar to the current study (1.36 µM). Additionally, different effect degrees were reported for late exposure scenarios ranging from only a 40% reduction at higher test concentrations (40 µM) (Parng et al. 2002) to a complete ISV inhibition at 2 µM (Panneerselvan and Ragunathan 2018).

Taken together, this work provides an integrated method for the automated visualization and quantification of ISV angiogenesis, including optimization of exposure windows to enhance the likelihood of detecting specific defects in vessel development in exposed zebrafish embryos. Within this study, due to the initial lack of annotated images and pre-trained models, ISVs were annotated manually. However, as a result of the study annotated images are now available for training models for a more automatic assessment using e.g. the FishInspector software. The subtraction approach and trained models can be applied in subsequent studies to address a variety of research questions including drug development and the identification of chemicals that pose a risk to the developing vascular system. Since fish embryos are considered as alternative approach to animal testing in many countries (Strähle et al. 2012), this method is compliant to the 3R concept (replacement, reduction and refinement of animal tests) in hazard and risk assessment of chemicals. Because developmental toxicity testing is required to perform hazard and risk assessment decisions for specific chemical classes, like impairment of the vascular network, this method is an easy to use high-content assay to predict developmental toxicity. Using the automated image acquisition and quantitative assessment of annotated morphological features we demonstrated that it is possible to relate effects on angiogenesis to other morphological endpoints. To identify other potential angiogenic compounds, this method can be applied to elucidate other environmental chemicals or teratogenic compounds as described in (McCollum et al. 2017; Tal et al. 2014). Furthermore, the method can be extended to include other endpoints relevant for the (functional) assessment of cardiovascular effects, such as blood flow velocity. The developed approach also provides the basis for subsequent studies to identify potential molecular biomarkers of anti-angiogenic processes by providing experimental conditions with high specificity for the detection of impaired angiogenesis.

### Supplementary Information

Below is the link to the electronic supplementary material.

Supplementary file1 (ZIP 1160360 KB)

Supplementary file2 (PDF 593 KB)

## Data Availability

All images and videos used for this publication can be found at the BioImage Archive S-BIAD954 (https://www.ebi.ac.uk/biostudies/bioimages/studies/S-BIAD954). Software and Workflows to perform data analyzing is available under https://git.ufz.de/automated-fish-embryotest/zebrafish-embryo-crc/zebrafish-isv. Supplementary files contain all results of quantitative image data.
