# Supplementary material for: Analysis of vascular disruption in zebrafish embryos as an endpoint to predict developmental toxicity
Source: Arch Toxicol. 2023 Dec 21;98(2):537–49. doi: 10.1007/s00204-023-03633-x (PMC10794345; doi:10.1007/s00204-023-03633-x)
Supplement: Supplementary file 2 — Supplementary file2 (PDF 593 KB) [file 204_2023_3633_MOESM2_ESM.pdf]

Supplement

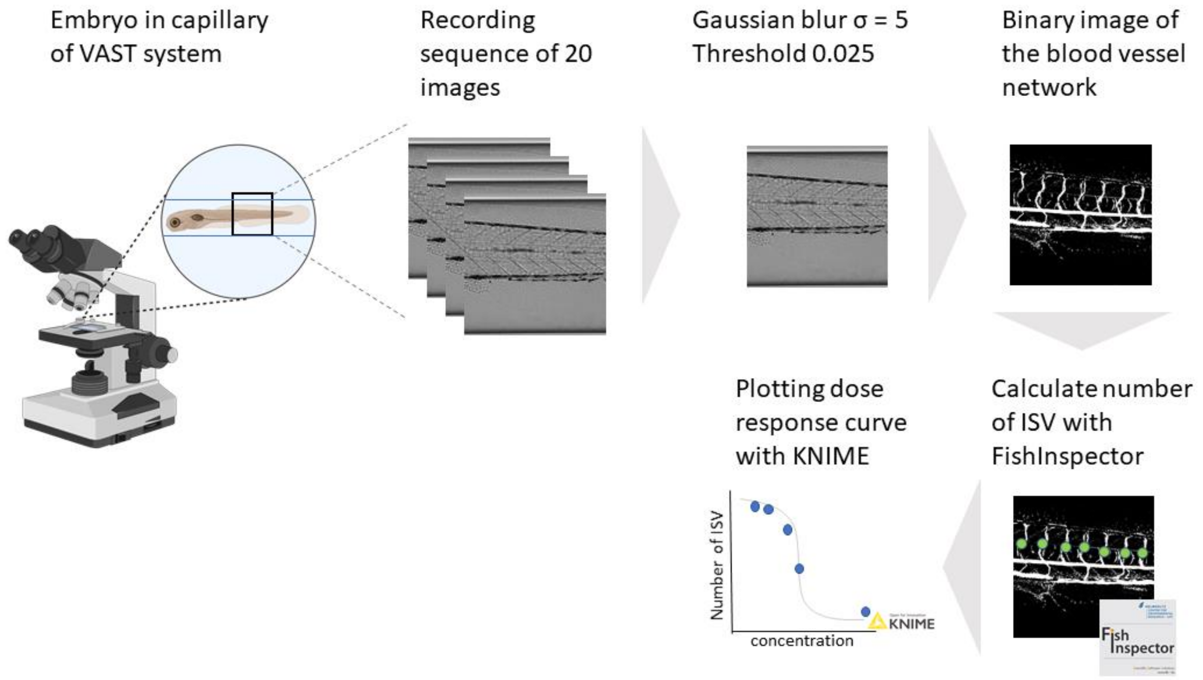

SI 1 How to convert the image series into a video to visualize the blood vessel system by applying a video subtraction approach. While imaging process using the VAST system, a series of 20 images was recorded. Briefly, each video frame was subtracted from the average image of the whole image sequence and converted to a binary image by applying a Gaussian blur (σ = 5) and a threshold of 0.025 to obtain optimal contrast in binary images. Subsequently, the sum of all binary images was calculated. Due to movement of the blood cells the subtraction approach removed all parts from the image which were not moving resulting in an image of the blood vessels. This binary image of the blood vessel network in the zebrafish embryo tail was then processed with the FishInspector annotation software. By positioning a 113 pixel diameter circle, the branches of ISVs were labelled manually. The number of ISVs was obtained by application of a KNIME workflow to FishInspector annotation files. Subsequently concentration–response analysis was conducted

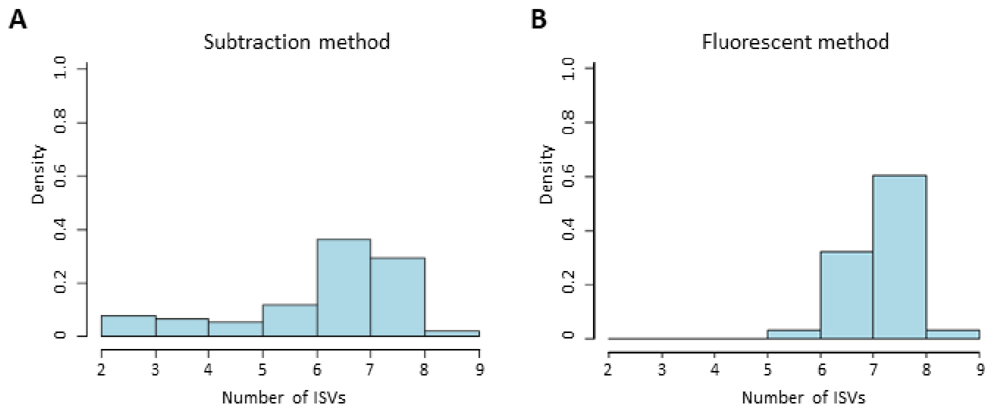

SI 2: Comparison of control variability of Tg(kdr:EGFP) strain. (A) analyzed as subtraction method (mean 6.6 ± 1.6 ISVs, n = 91) or (B) as fluorescent method (mean 7.6 ± 0.6 ISVs, n = 86).

SI 3: Sensitivity comparison between Fluorescence and subtraction-based analyses of ISV numbers after SU4321 treatment.

Method	EC50	SE	95% confidence	EC10	SE	95% confidence
Fluorescence images	> 10 µM	NA	NA	1.17 µM	0.17 µM	± 4.3 µM
Subtraction images	2.98 µM	0.33 µM	2.98 µM	0.72 µM	0,23 µM	4.88 µM

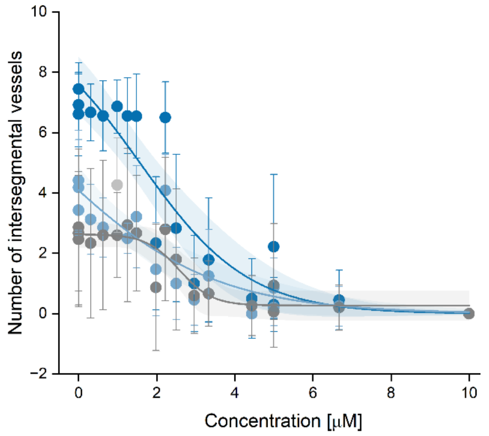

	EC50	SE	95% confidence
All ISV	2.98 µM	0.33 µM	2.98 µM
vISV	0.75 µM	0.43 µM	0.17 µM
aISV	2.58 µM	0.25 µM	3.12 µM

SI 4: Dose-response curves of all visible all ISVs (blue, EC50 2.98 µM) and only vISVs (light blue, EC50 0.75 µM) or aISVs (grey, EC50 2.58µM) after SU4312 exposure. It is possible to distinguish between aISVs and vISVs using the subtraction-based method.

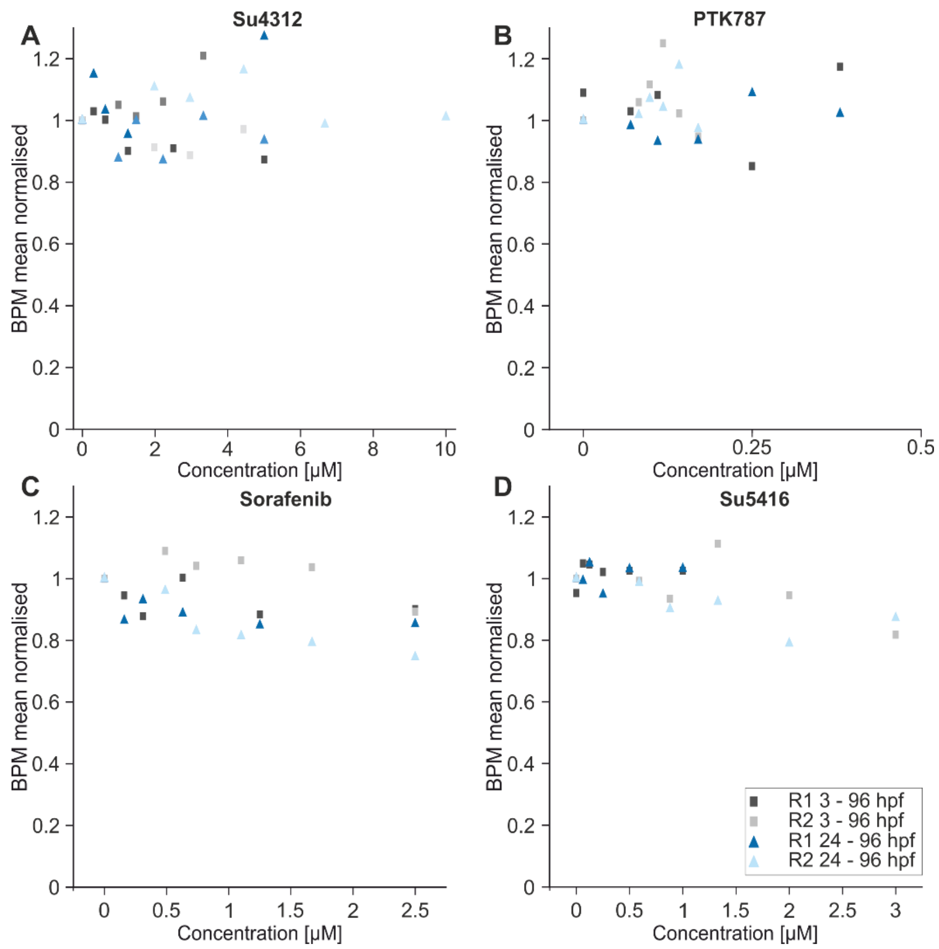

SI 5: Concentration-dependent heart rate assessment as indicator of cardiotoxicity. of the heart rate for exposure to (A) SU4312, (B) SU5416, (C) Sorafenib and (D) PTK787 was obtained from video analysis and is given as the mean beats per minute (BPM) of 96 hpf zebrafish embryos. Different color shades indicate replicates. Based on tukey trend test and AIC estimation no concentration-dependency was observed. No change between early (grey) or late (blue) exposure scenario

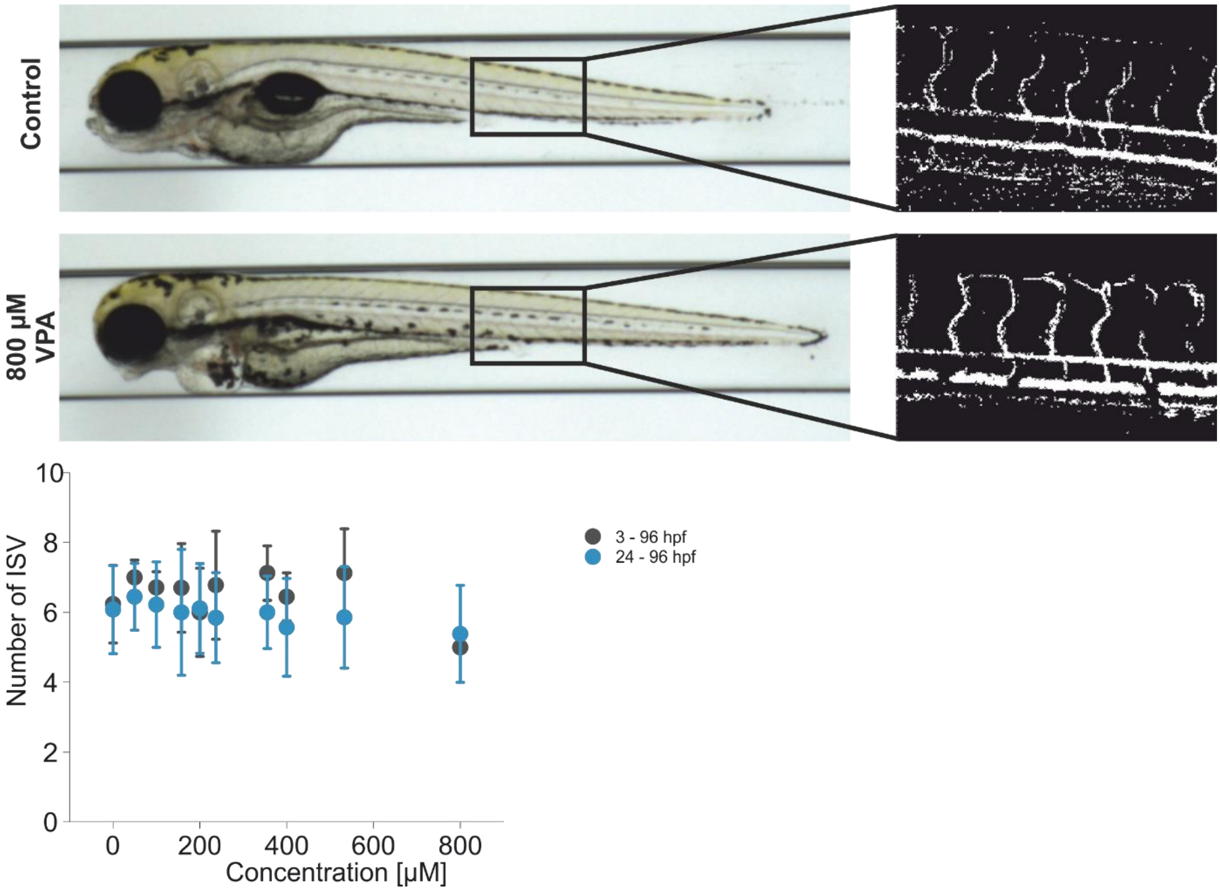

SI 6: Subtraction images of negative control VPA. Embryos were exposed to (A) vehicle (0.01% DMSO) or (B) 800 µM VPA at 24 hpf. At 96 hpf, larvae were visualized. The tail region of embryos was imaged using the video subtraction method to investigate teratogenic substances, which does not influence ISV formation.

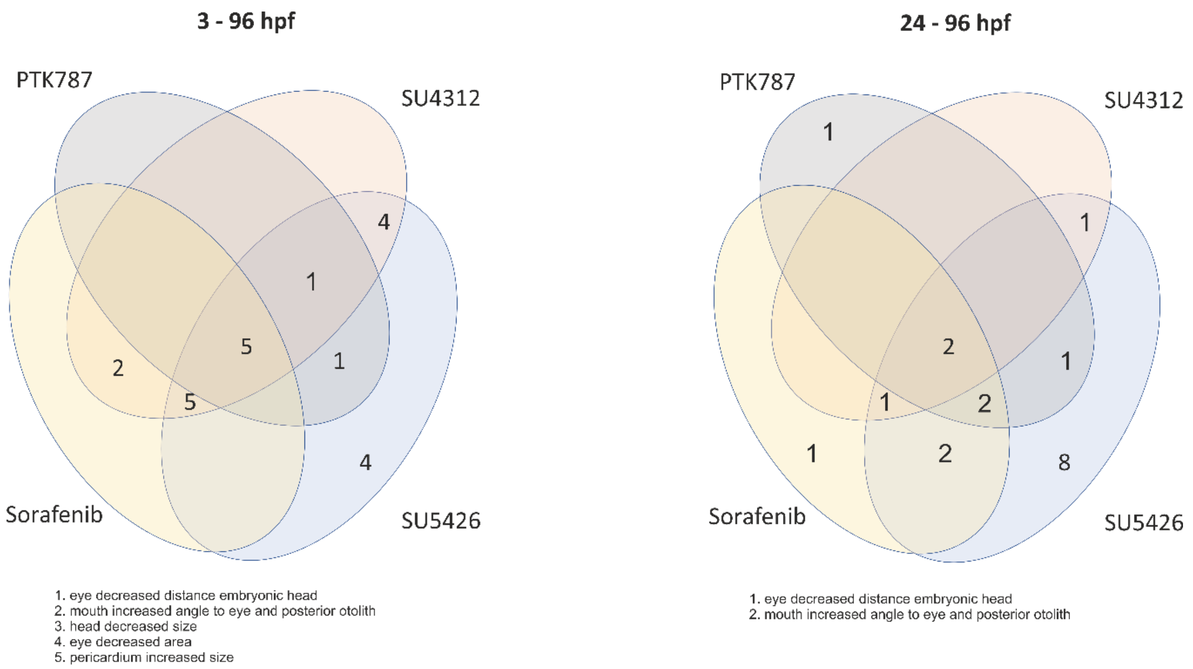

SI 7 Venn Diagram to compare the number of occurred morphological effects (indicated by a corresponding EC_{50}) shared between the different test compounds
